# Cripto-1 acts as a functional marker of cancer stem-like cells and predicts prognosis of the patients in esophageal squamous cell carcinoma

**DOI:** 10.1186/s12943-017-0650-7

**Published:** 2017-04-21

**Authors:** Qiang Liu, Xiang Cui, Xi Yu, Bai-Shi-Jiao Bian, Feng Qian, Xu-gang Hu, Cheng-dong Ji, Lang Yang, Yong Ren, Wei Cui, Xia Zhang, Peng Zhang, Ji Ming Wang, You-hong Cui, Xiu-wu Bian

**Affiliations:** 1 Institute of Pathology and Southwest Cancer Center, and Key Laboratory of Tumor Immunopathology of Ministry of Education of China, Southwest Hospital, Third Military Medical University, Chongqing, 400038 China; 2 Breast Disease Center, Southwest Hospital, Third Military Medical University, Chongqing, China; 3 Department of General Surgery, Southwest Hospital, Third Military Medical University, Chongqing, China; 4 Cancer and Inflammation Program, Center for Cancer Research, National Cancer Institute, Frederick, MD USA

**Keywords:** Cripto-1, Esophageal squamous cell carcinoma, Cancer stem-like cells, Metastasis, Prognosis

## Abstract

**Background:**

Esophageal squamous cell carcinoma (ESCC) is highly malignant with highly invasive and metastatic capabilities and poor prognosis. It is believed that the ESCC cancer stem-like cells (ECSLCs) are critical for tumorigenicity, invasion and metastasis of ESCC. However, the properties of ECSLCs vary with different markers used in isolation, so that new and more effective markers of ECSLCs need to be identified. This study aimed to estimate the potentiality of Cripto-1 (CR-1) as an ECSLC surface marker and investigate the clinical significance of CR-1 expression in ESCC.

**Methods:**

ESCC cells with CR-1 ^high^ or CR-1^low^ were obtained by flow cytometry then their self-renewal capability and tumorigenicity were compared by colony and limiting dilution sphere formation analysis in vitro and xenograft in nude mice in vivo, respectively. Knockdown of CR-1 expression in ESCC cells was conducted with short hairpin RNA. Cell migration and invasion were examined by scratch test and matrigel transwell assay, respectively. Metastatic capability of ESCC cells was assayed by a mouse tail vein metastasis model. The levels of CR-1 expression in cancerous and paired adjacent normal tissues were assessed by IHC and qRT-RCR.

**Results:**

CR-1^high^ subpopulation of ESCC cells isolated by FACS expressed high level of genes related to stemness and epithelial-mesenchymal transition (EMT), and possessed high capacities of self-renewal, tumorigenesis, invasion and metastasis. Suppression of CR-1 expression significantly reduced the expression of stemness- and EMT-related genes and the capabilities of self-renewal in vitro, tumorigenicity and metastasis in vivo in ESCC cells. In the clinical ESCC specimens, the expression levels of CR-1 in cancerous tissues were positively correlated to TNM stage, invasive depth, and lymph node metastasis. Cox regression analysis indicated that CR-1 was an independent indicator of prognosis. The expression of CR-1 was found overlapping with aldehyde dehydrogenase 1A1 (ALDH1A1), an intracellular marker for ESCLCs, in ESCC cell lines and specimens.

**Conclusions:**

CR-1 is a functional and cell surface ECSLC marker, and an independent prognostic indicator as well as a potential therapeutic target for ESCC.

**Electronic supplementary material:**

The online version of this article (doi:10.1186/s12943-017-0650-7) contains supplementary material, which is available to authorized users.

## Background

Esophageal squamous cell carcinoma (ESCC), the dominant subtype of esophageal cancer, is the fourth leading cause of cancer-related deaths in China [1]. ESCC has remarkable geographic distribution, with distinctly high incidences and mortality rates especially in China and other Asian countries [2]. As a malignant tumor with highly invasive and metastatic capacities, ESCC has a low 5-year survival rate after surgery and chemotherapy. Better understanding the underlying cellular and molecular mechanisms is urgently needed in order to develop novel and effective therapeutics.

The cancer stem-like cells (CSLCs), or tumor-initiating cells, are a small subpopulation of tumor cells that play an important role in tumorigenicity, invasion and metastasis [3], and are considered as one of the most important therapeutic targets. Up to now, our and other groups have isolated and characterized ESCC cancer stem-like cells (ECSLCs) using aldehyde dehydrogenase 1A1 (ALDH1A1) [4], CD44 [5], p75^NTR^ [6, 7] and CD90 [8] as markers. However, the properties of ECSLCs vary with different markers used in isolation, so that new and more effective markers of ECSLCs need to be identified.

Human cripto-1 (CR-1), a glycosylphosphatidylinositol-linked (GPI-linked) anchor membrane protein [9], also known as the teratoma-derived growth factor 1 (TDGF-1), belongs to the EGF-CFC family of growth factor-like molecules. CR-1 plays very important roles in the early embryonic development; however, is absent or only weakly expressed in normal adult cells [10]. A growing body of evidence has been emerging that CR-1 is overexpressed in several types of cancer, including human embryonal carcinoma [11], and colon, brain, liver, breast, lung cancers [12–16]. Furthermore, it has been reported that CR-1 positive cells exhibit stem cell-like characteristics in human melanoma and colorectal cancer [17, 18].

In this study, we investigated the stem cell-like characteristics of CR-1 positive ESCC cells and the clinical relevance of CR-1 expression in human ESCC samples. Our results indicate that CR-1 is a functional CSLC marker and an independent indicator of prognosis as well as a potential target for therapeutics in ESCC.

## Methods

### Cell lines and culture

Human ESCC cell lines EC109 and TE-1 were obtained from the cell bank of Chinese Academy of Sciences (CAS), and maintained in Dulbecco’s modified Eagle’s medium (DMEM) (Gibco/Life Technologies, Carlsbad, CA) supplemented with 10% fetal calf serum (FBS) (Gibco/Life Technologies) at 37 °C, 5% CO_2_ in moist atmosphere.

### Colony formation assay

Colony formation assay was performed as previously described [4]. Briefly, cells were seeded in 24-well plates at the density of 50 cells per well in 200 μL DMEM supplemented with 10% FBS. The medium was changed every three days. After three weeks, the cell colonies were fixed with 4% paraformaldehyde for 20 min and then stained with crystal violet for 15 min. After washing three times with PBS, the number of colonies larger than 1 mm was counted in each group.

#### Limiting dilution assay

Limiting dilution assay was performed to measure the frequency of ESCLCs as previously described [4]. Briefly, serial twofold dilutions of CR-1 ^high^ and CR-1 ^low^ cells (from 100 to 0 cells) were sorted into ultra-low 96-well plates (Costar, USA) with 10 wells per dilution, respectively. The cells were cultured in 100 mL of serum-free Dulbecco’s modified Eagle’s medium (DMEM)/F12 medium supplemented with EGF (20 ng/mL) and bFGF (20 ng/mL) at 37 °C and 5% CO2. Twenty microliters of fresh medium were added to each well every 3 days. Spheres were counted at day 7. Fraction of wells without spheres (log 2) (y-axis) was plotted against the number of cells plated per well (x-axis).

### In vitro migration and invasion assays

Scratch wound assay was carried out to measure distance of cell migration. ESCC cells (5 × 10^5^) were cultured in 200 μL DMEM medium containing 10% FBS in 24-well plates until monolayer cell reached confluence. Then culture medium was replaced with serum-free DMEM medium. Wounds were created by scratch with thin pipette tips. Wound healing processes were imaged at 0 and 24 h using a reverse phase microscope. Cell migration distances were obtained from the images by using ImageJ software (NIH, Bethesda, MD). For invasion assay in vitro, transwell chambers (Millipore) were coated with 10 μL of DMEM diluted Matrigel™ (1:1, BD Biosciences) and ESCC cells suspended in serum-free medium (200 μL containing 3 × 10^4^ cells) were added to the upper chamber. The medium (600 μL) contained 10% FBS (Gibco) was added to the lower chamber. After 24 h incubation, the non-invasive cells in upper chamber were carefully removed with a cotton swab. The migration cells that passed through the membrane and adhered to the lower surface of the membrane were fixed with 4% paraformaldehyde for 15 min, and then stained with crystal violet for 5 min. Invasion cells were counted in five different fields under light microscope.

### Xenograft in nude mice

The nude mice at age from 4 to 5 weeks and weighing range between 17 and 20 g were provided by the Experimental Animal Center of Third Military Medical University, and were maintained in no-specific pathogen environment. The animal experiments were approved by the Third Military Medical University Animal Ethics Committee. ESCC shCR1 and Mock cells were injected subcutaneously into axilla of nude mice with implantation of 2 × 10^4^ and 2 × 10^5^ cells per mouse, respectively (*n* = 6). Sorted CR-1^high^ and CR-1^low^ cells were performed as abovementioned at 1 × 10^4^ cells, respectively (*n* = 6). At the end of sixth week, mice were euthanized and xenograft tumors were removed and weighted. Immunohistochemical staining was used to evaluate xenografted tumor proliferation with rabbit anti-human Ki67 polyclonal antibody (Beijing Zhongshan Jinqiao Company).

### In vivo metastasis assay

shCR1 and Mock EC109 cells were suspended in 10 μL PBS and injected into mice via tail vein at 2 × 10^5^ cells per mouse, respectively (*n* = 6). All mice were euthanized at the end of the sixth week after the injection and the tumor metastasis nodules in the lung were examined.

### Patients and tissue specimens

Tumors and their corresponding adjacent normal tissues as well as metastatic lymph nodes were obtained from 138 ESCC patients who had not received chemotherapy or radiotherapy before operation in Southwest Hospital of the Third Military Medical University from 2006 to 2007. Follow-up information of 138 patients was available for a period of minimal 5 years. All samples were confirmed by pathological examination according to the American Joint Committee on Cancer (AJCC) Cancer Staging Manual (7th edition). Written informed consents for the biological studies were obtained from the patients or their guardians. All experiments were approved by Ethics Committee of Southwest Hospital.

### Immunohistochemistry (IHC)

Immunohistochemical staining was performed with Dako REAL™ EnVision™ detection System (Code K5007; Dako, Glostrup, Denmark) as previously described [19]. Briefly, formalin-fixed, paraffin-embedded esophageal cancer sections (4 mm) were pretreated by 0.3% H2O2 and antigen retrieval was performed according to the manufacturer’s instruction. The slides were then incubated with a rabbit anti-human CR-1 polyclonal antibody (1:150; ab19917, Abcam, UK) at 4 °C overnight. The secondary antibody was added for incubation at 37 °C for 30 min. The cells with brown color in the membrane and cytoplasm were counted as positive cells.

The CR-1 expression level was reported by multiplication of staining density and average percentage of positive cells. The Staining intensity was scored with “0” (no staining), “1” (weakly positive), “2” (moderately positive), and “3” (strongly positive). The staining average percentage of positive cells was scored as:0 = 0%, 1 = 1–25%, 2 = 26–50%, 3 = 51–75%, and 4 = 76–100%. All slides were evaluated independently by two pathologists in a double-blinded manner. It was defined as low expression if calculation of the score less than 6, the cut-off derived from X-tile analysis [20], otherwise they were defined as high expression.

### Statistical analysis

Kaplan-Meier survival plots and log-rank statistics were used to evaluate the survival of patients. Cox multivariate regression was used to analyze multiple factors affecting the prognosis. Pearson *χ* ^2^ test was used to analyze the relationship between CR-1 expression and clinico-pathological parameters. Data were expressed as the mean ± standard deviations (S.D.) and the statistical significance between testing and control groups was analyzed with SPSS 18.0 statistical software. The cut-off of IHC score was analyzed by X-tile [20]. When two groups were compared, the unpaired Student’s *t* test was used. *p* < 0.05 was considered statistically significant.

### Quantitative RT-PCR, Western blotting, Flow cytometry, Silencing CR-1 with shRNA, and Immunofluorescence staining

See “supplementary materials and methods” in the Additional file 1. Primers used in this study were listed in Additional file 1: Table S1, and the sequences of the shRNA targeting CR-1 were listed in Additional file 1: Table S2.

## Results

### CR-1^high^ cells isolated from ESCC cells possess CSLC properties

**Fig. 1:**
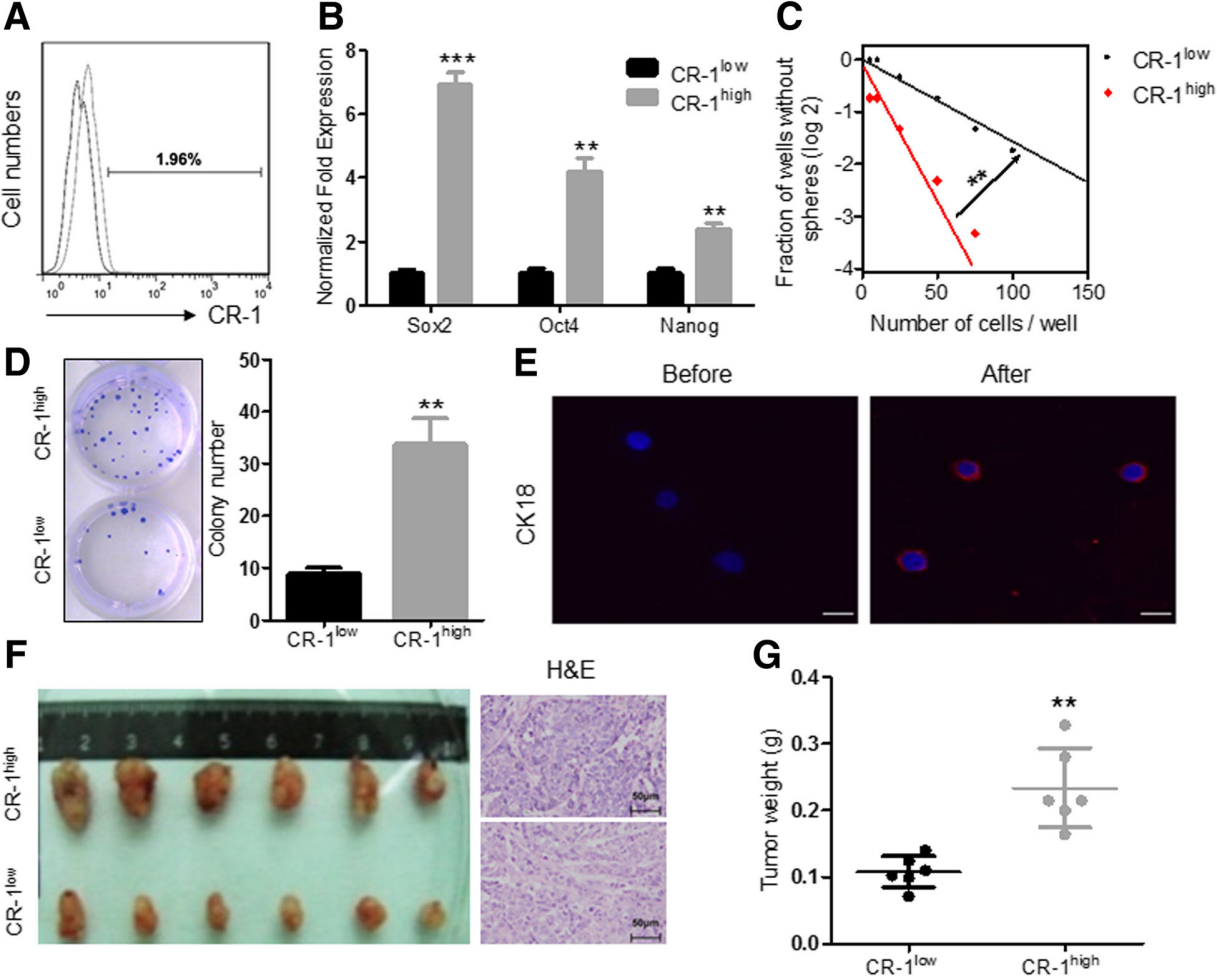
CR-1^high^ cells possess stemness properties compared to CR-1^low^ cells. **a** Representative flow cytometric histogram of the percentage of CR-1^high^ cells in EC109 cells. **b** qRT-PCR analysis showed that CR-1 ^high^ cells highly express stemness-related transcription factors Sox2, Oct4 and Nanog. **c** Limiting dilution assay showed that CR-1 ^high^ cells possessed higher capability of sphere formation than CR-1 ^low^ cells. **d** Colony formation of CR-1^high^ cells was stronger than CR-1^low^ cells (*left*: crystal violet staining, *right*: quantitative analysis). **e** Confocal microscopic analysis revealed that CK18 expression was up-regulated in CR-1^high^ cells after inducing differentiation for 7 days. **f** Subcutaneously xenografted tumor model indicated that tumors derived from CR-1^high^ cells were larger than those from CR-1^low^ cells (*left*). The histological origination of xenografts was confirmed by H&E staining (*right*). **g** The weight of the xenografts derived from CR-1^low^ and CR-1^high^ cells. Bar, 10 μm; * indicates *p* < 0.05; ** indicates *p* < 0.01

We first examined the expression of CR-1 in two human ESCC cell lines EC109 and TE-1, and found that both of the two cell lines expressed CR-1 at mRNA and protein levels, and the expression level of EC109 cells was approximately 2 ~ 3 fold higher than that of TE-1 cells (Additional file 1: Figure S1A and S1B). With fluorescence activated cell sorting (FACS), two distinct subpopulations, CR-1^high^ and CR-1^low^, were isolated from the cell lines. The percentage of CR-1^high^ cells was 1.96 ± 0.5% (*n* = 9) in the EC109 cells (Fig. 1a). We then assessed the cancer stem like cell (CSLC) properties of CR-1^high^ cells from the aspects of stemness-related gene expression, self-renewal ability, differentiation potential and tumorigenecity [3]. Sorted CR-1^high^ cells expressed higher levels of important stemness-related transcription factors Sox2, Oct4 and Nanog than that of CR-1^low^ cells (Fig. 1b). Quantitative tumorsphere and colony formation assays were used to estimate the self-renewal ability of the cells in vitro. As shown in Fig. 1c, CR-1^high^ EC109 cells exhibited stronger capacity of sphere formation as compared to CR-1^low^ cells by limiting dilution assay (*p < 0.01*). Colony formation assay revealed that CR-1^high^ cells had stronger capacity of plate clone formation as compared to CR-1^low^ cells (33.75 ± 4.90 vs. 9.00 ± 1.00, *p* < 0.001, Fig. 1d). The CR-1^high^ cells also showed higher differentiation potential as indicated by the expression of a differentiation marker CK18 (Fig. 1e). The in vivo tumorigenicity studies were conducted in subcutaneously xenografted nude mice with 1 × 10^4^ cells per mouse and harvested the tumor masses at the end of 6 weeks after injection. The tumors derived from CR-1^high^ cells were markedly larger in size than that of CR-1^low^ (Fig. 1f), and the average weight of xenografts derived from CR-1^high^ and CR-1^low^ cells was 0.23 g ± 0.06 g and 0.11 g ± 0.02 g, respectively (*p* = 0.001, Fig. 1g). These results indicate that CR-1^high^ ESCC cells possess CSLC properties and CR-1 could be a specific CSLC marker for ESCC.

### Silencing CR-1 expression significantly reduces stemness of ESCC cells

**Fig. 2:**
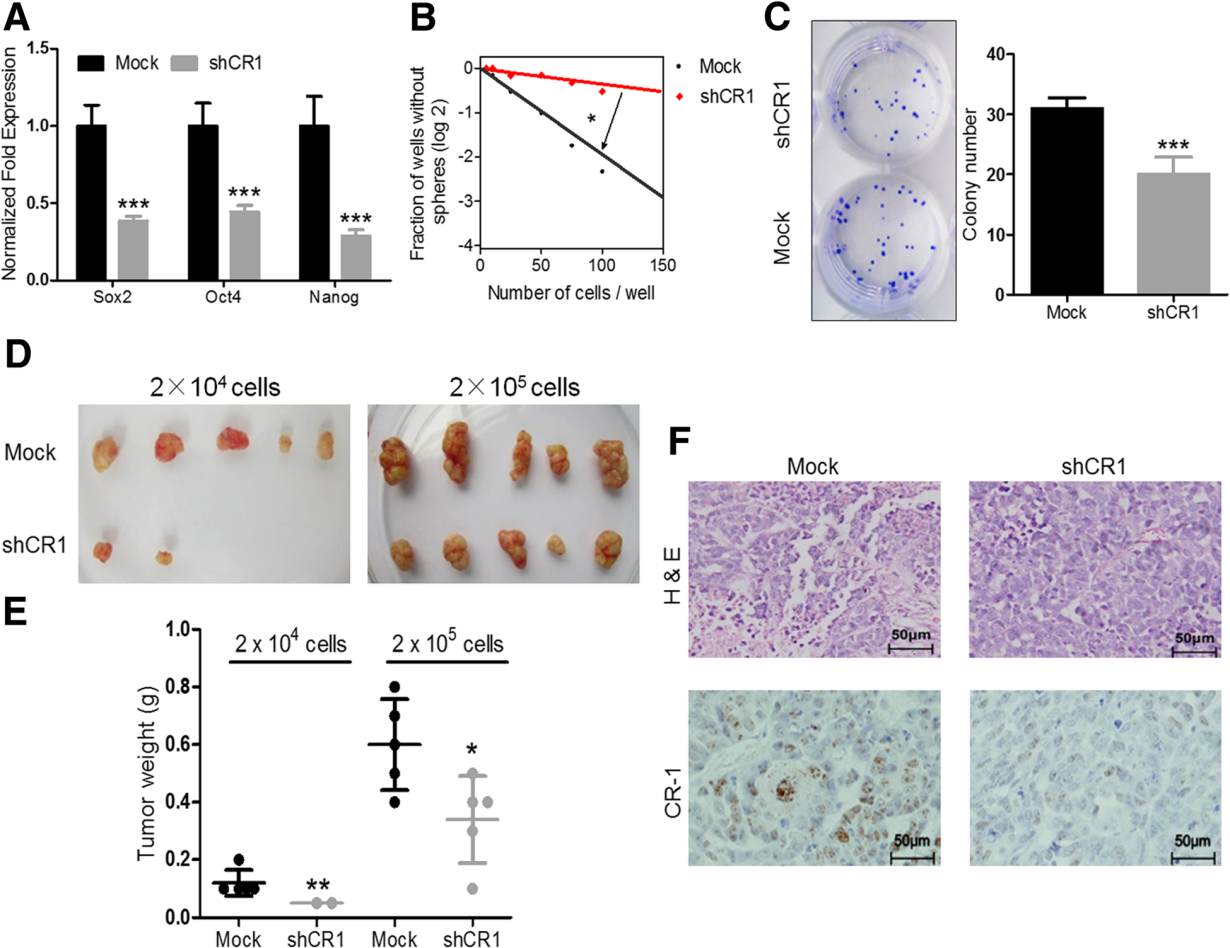
CR-1 knockdown significantly represses the self-renewal and tumorigenicity of EC109 cells. **a** CR-1 knockdown (shCR1) dramatically decreased the expression of stemness-related transcription factors Sox2, Oct4 and Nanog. **b** Limiting dilution assay showed that CR-1 knockdown markedly suppressed sphere formation. **c**. CR-1 knockdown significantly repressed colony formation. **d** Representative images showed that CR-1 knockdown decreased tumor formation in a xenografted tumor mouse model. **e** Quantitative analysis of the weight of xenografts formed by EC109 cells (2 × 10^4^ cells and 2 × 10^5^ cells, respectively). **f** H&E staining confirmed the origination of xenografts and IHC staining confirmed that xenografts derived from shCR-1 cells express less CR-1 compared to Mock cells. * indicates *p* < 0.05; ** indicates *p* < 0.01

To confirm the functional roles of CR-1 in ECSLCs, we silenced CR-1 expression with lentivirus carrying CR-1 shRNA in EC109 and TE-1 cells (shCR1-EC109 and shCR1-TE-1 cells). The silencing efficiency was demonstrated by western blotting assay (Additional file 1: Figure S1C and S1D). After CR-1 knockdown, the expressions of Sox2, Oct4 and Nanog were significantly down-regulated in shCR1-EC109 cells (Fig. 2a). In contrast to Mock cells, shCR1-EC109 cells exhibited significantly decreased sphere forming frequency by limiting dilution assay (*p < 0.05*, Fig. 2b) and clonogenic capability (31.00 ± 1.83 vs. 20.00 ± 2.94, *p* = 0.0007, Fig. 2c). The xenograft assays showed that 2 × 10^4^ Mock cells were sufficient to form tumors within 6 weeks in all implanted nude mice (5/5), whereas same number of shCR1-EC109 cells resulted in only 40% (2/5) tumor formation with smaller size and lower weight (Fig. 2d and e, Additional file 1: Table S3). H&E staining confirmed that the xenografts were derived from ESCC cells and IHC staining showed that the level of CR-1 expression in xenografts derived from Mock cells was higher than that of the shCR1 cells (Fig. 2f). Silencing CR-1 expression in TE-1 cell line resulted in similar results as that obtained from EC109 cell line (Additional file 1: Figure S2, Table S3). These results suggest that CR-1 may not only act as a cell surface marker but also function in regulating stemness of ECSLCs.

### CR-1^high^ cells exhibits highly invasive and metastatic properties in association with an epithelial-mesenchymal transition (EMT) phenotype

**Fig. 3:**
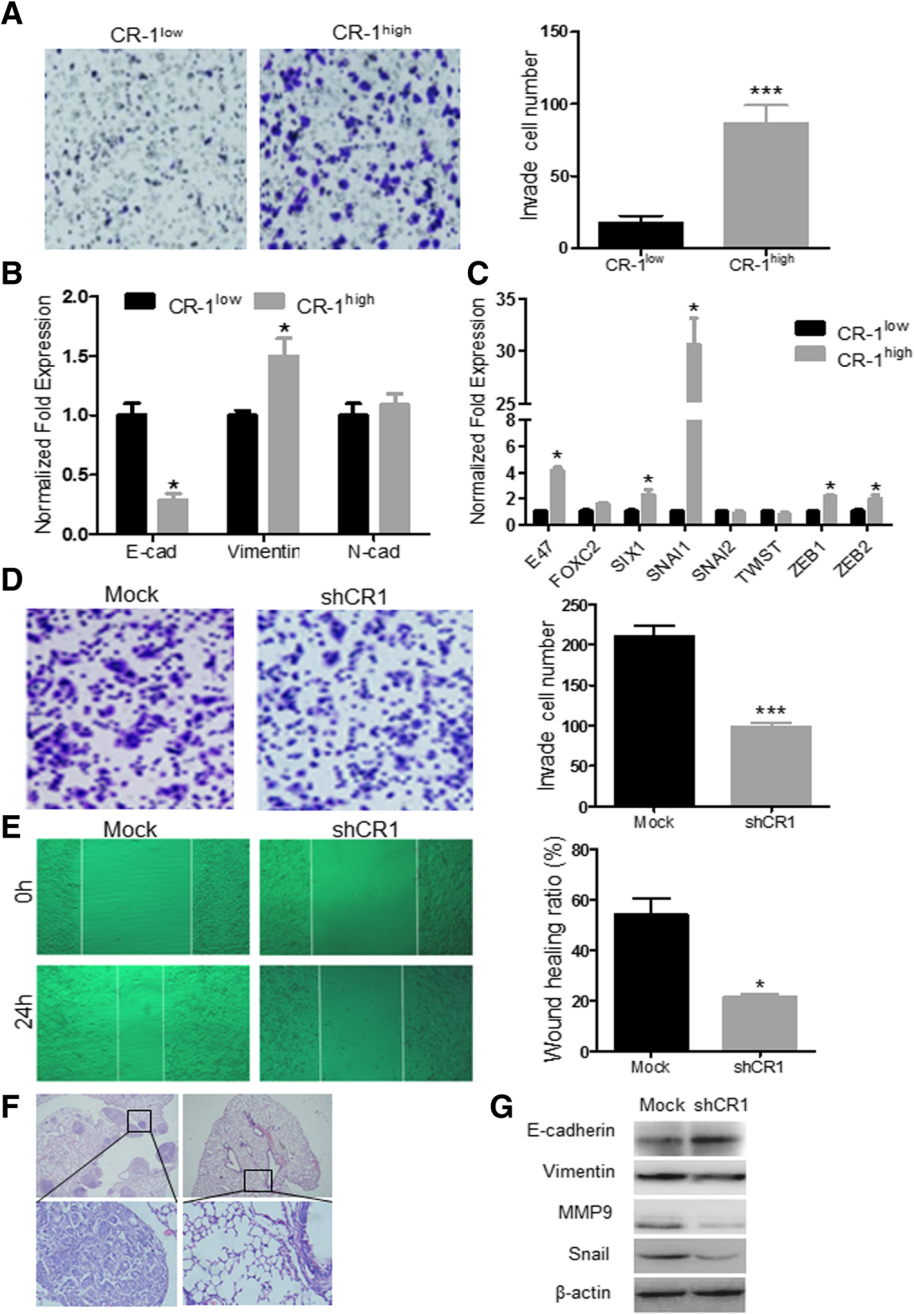
CR-1 is associated with the invasion and metastasis of EC109 cells in vitro and in vivo. **a** Transwell chamber invasion assay revealed that the invasion capacity of CR-1^high^ cells was stronger than that of the CR-1^low^ cells. **b** qRT-PCR analysis of the expressions of E-cadherin and vimentin in CR-1 ^high^ and CR-1 ^low^ cells. **c** qRT-PCR analysis of differential expression of EMT-related transcription factors in CR-1 ^high^ and CR-1 ^low^ cells. **d** Silencing CR-1 expression significantly decreased the invasion of EC109 cells in vitro. **e** Wound healing experiments showed that CR-1 knockdown significantly reduced the capacity of migration in EC109 cells. **f** CR-1 knockdown suppressed the metastatic potential of EC109 cells in nude mice. **g** Western blot assay showed that down-regulation of CR-1 results in up-regulation of E-cadherin and down-regulation of vimentin, snail and MMP9 in EC109 cells. * indicates *p* < 0.05; *** indicates *p* < 0.001

Since the invasion and metastasis are the important biological characteristics of ESCC, we then evaluated the roles of CR-1^high^ ECSLCs in these malignant behaviors of ESCC. The in vitro invasion experiments showed that CR-1^high^ cells possessed significantly higher ability of invasion than that of CR-1^low^ cells (87 ± 12.3 *vs* 17 ± 5.6, *p* < 0.001, Fig. 3a), implying that CR-1^high^ ECSLCs might be the major player in the invasion and metastasis of ESCC. EMT has been believed to associate with early steps of invasion and metastasis of epithelial origin cancer cells and generation of CSLCs [21], we then examined the differential expression of EMT characteristic molecules and EMT-related transcription factors in CR-1^high^ and CR-1^low^ cells by qRT-PCR. The CR-1^high^ cells expressed lower level of E-cadherin but higher level of vimentin compared to CR-1^low^ cells (*p* < 0.05, Fig. 3b). Reduced E-cadherin expression is considered to be a fundamental event in EMT [22]. During the EMT-related transcription factors, snail binds to E-cadherin promoter and represses its transcription, whereas factors such as Twist, Goosecoid and FOXC2 repress E-cadherin indirectly. CR-1^high^ cells expressed about 30 folds higher of snail, and 1.6 ~ 4.1 folds higher of E47, Foxc2, SIX1, ZEB1 and ZEB2 than that of CR-1^low^ cells (Fig. 3c). These results indicate that CR-1 is associated with the mesenchymal-like characteristics in CR-1^high^ EC109 cells.

We further evaluated the effect of silencing CR-1 expression on the migration and invasion capacities of EC109 cells in vitro. Results from transwell matrigel invasion assay showed that knockdown of CR-1 significantly inhibited the invasion capacity of EC109 cells (Fig. 3d). The number of invasive cells from shCR1 and Mock cells were 99 ± 4.8 and 211 ± 12.3, respectively (*p* < 0.001). The percentage of wound healing ratio by Mock cells and shCR1 cells were 54.1 ± 6.3% and 21.4 ± 1.3%, respectively (*p* = 0.011, Fig. 3e). We further evaluated whether knockdown of CR-1 expression suppressed metastasis in vivo. shCR1-EC109 and Mock cells were injected to nude mice by tail vein injection with 1 × 10^4^ cells/mouse. Six weeks after injection, the lungs, mediastinal lymph nodes, brain, livers, and kidneys of the transplanted mice, were carefully examined and the metastatic foci were found only in the lungs. The frequency of metastasis to lungs was much lower in nude mice implanted with shCR1 cells as compared to Mock cells (*p* = 0.030, Fig. 3f, Additional file 1: Table S4). Knockdown of CR-1 expression also resulted in up-regulation of the E-cadherin and down-regulation of vimentin and Snail in EC109 cells (Fig. 3g). Similar results were obtained from TE-1 cells (Additional file 1: Figure S3, Table S4). These results suggest that CR-1^high^ ECSLCs may play a crucial role in the invasion and metastasis of ESCC, which are associated with the EMT phenotype regulated by CR-1.

In addition, we investigated the expression of matrix metalloproteinases (MMPs), which promote invasion and metastasis of tumor cells by degrading the extracellular matrix, and found that silencing of CR-1 expression significantly down-regulated MMP9 expression at both mRNA and protein levels (Fig. 3g and Additional file 1: Figure S4), whereas the expression of MMP2 and MMP7 was not been affected. These results suggest that the high invasive and metastatic capacities of CR-1^high^ ECSLCs are associated not only with up-regulating EMT phenotype but also with up-regulating MMP9 expression.

### Elevated CR-1 expression in ESCC specimens is correlated to poor prognosis of the patients

**Fig. 4:**
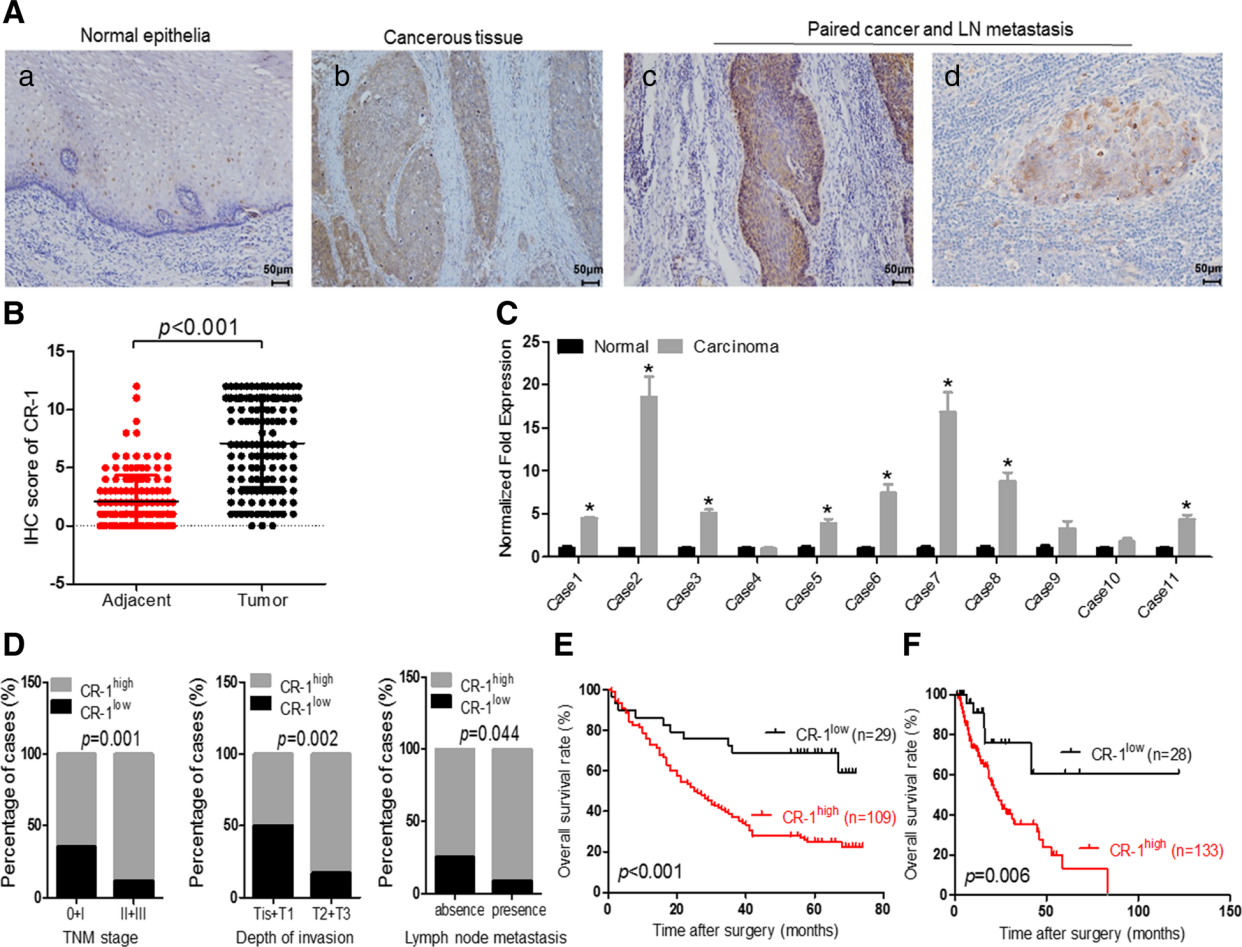
Correlation of CR-1 expression with clinical pathological parameters and overall survival rates in ESCC patients. **A** (*a*) Representative immunohistochemical images showed few CR-1^high^ cells that were localized in the basal layer of normal squamous epithelium. (*b*) High level of CR-1 protein abundance in ESCC tissues. (*c*, *d*) CR-1 highly expressed in both primary tumor and the corresponding metastatic lymph node. **B** The IHC score of CR-1 in cancerous tissues was higher than that of adjacent normal tissues. **C** Quantitative analysis of CR-1 mRNA in 11 fresh surgical tumor specimens and the adjacent normal tissues showed that CR-1is highly expressed in cancerous tissues. **D** CR-1 expression was significantly associated with TNM stage, depth of invasion, and lymph node metastasis. **E** Kaplan-Meier estimation of correlation between overall survival and CR-1 expression in ESCC patients indicated that the patients with CR-1^high^ had shorter overall survival time than those with CR-1^low^. **F** Survival analysis of ESCC patients from TCGA dataset indicated that patients with CR-1^high^ suffered poorer overall survival

To investigate the clinical significance of CR-1 expression in ESCC, IHC was performed on sections of ESCC specimens and paired adjacent normal tissues from 138 patients. The CR-1 staining was localized in the plasma membrane and cytoplasm of cells (Fig. 4A). The proportion of CR-1^high^ in cancerous tissues was markedly higher than that in paired adjacent normal tissues (69.6% vs. 30.4%, *p* < 0.001) (Additional file 1: Table S5). As shown in Fig. 4Aa, a representative image of adjacent normal squamous epithelia had only a few scattered CR-1^high^ cells in the basal layer. In contrast, strong CR-1 staining was observed in ESCC tumor tissues and metastatic lymph node (Fig. 4Ab-d). Semi-quantitatively, the IHC score of CR-1 was significantly higher in the cancerous tissues than that in the adjacent normal tissues (7.23 ± 3.88 vs., 2.12 ± 2.23, *p* < 0.001, Fig. 4B). In a separate set of samples, quantitative analysis of CR-1 mRNA in 11 fresh surgical tumor specimens and the adjacent normal tissues indicated that 10 out of 11 tumor specimens had high level of CR-1 expression (2 ~ 18 fold higher) as compared to their adjacent normal tissues (Fig. 4C). These results indicate that cancer cells highly express CR-1 in ESCC.

We further investigated the relationship of CR-1 expression with the clinico-pathological features and found that CR-1 expression was significantly associated with depth of invasion (*p* = 0.002), TNM stage (*p* = 0.001) and lymph node metastasis (*p* = 0.044, Fig. 4D), suggesting high invasive/metastatic capacity of CR-1^high^ cell subpopulation. There was no significant association between CR-1 expression and gender (*p* = 0.636), age (*p* = 0.106), and histological grade (*p* = 0.825). General summarization was presented in Table 1. Survival curves of 138 patients were analyzed using the Kaplan-Meier method. A significant correlation was found between high expression of CR-1 and decreased overall survival rate in ESCC patients (*p* < 0.001, Fig. 4E). The data obtained from TCGA dataset also indicated that patients with high expression of CR-1 suffered poorer overall survival than those with low expression of CR-1 (*p* = 0.006, Fig. 4F). Multivariate Cox-regression analysis showed that only the depth of tumor invasion (HR = 1.465, 95% CI = 1.655–6.395, *p* = 0.018) and the expression of CR-1 (HR = 3.253, 95% CI = 1.066–1.2.011, *p* = 0.001) were independent prognostic indictors in ESCC patients (Table 2). These results strongly suggest that CR-1^high^ predicts poor prognosis and the CR-1^high^ cell population has strong invasion and metastasis capacities.

**Table 1:** CR-1 expression was positively correlated with clinical pathologic parameter

Clinicopathological parameter	Case (%)	Expression of CR-1		*p* Value
		CR-1^low^ (%)	CR-1^high^ (%)	
Gender				0.636
Male	118(85.5)	24(82.8)	94(86.2)	
Female	20(24.5)	5(17.2)	15(13.8)	
Age (year)				0.106
≤ 62	66(47.8)	10(34.5)	56(51.4)	
> 63	72(52.2)	19(65.5)	53(48.6)	
Depth of invasion				0.002
T_is_ + T_1_	16(11.6)	8(27.6)	8(7.3)	
T_2_ + T_3_	122(88.4)	21(72.4)	101(92.7)	
TNM Stage				0.001
0 + I	54(39.1)	19(65.5)	35(32.1)	
II + III	84(60.9)	10(34.5)	74(67.9)	
Lymph node metastasis				0.044
Yes	34(24.6)	3(10.3)	31(28.4)	
No	104(75.4)	26(89.7)	78(71.6)	
Histological grade				0.825
G1	88(63.8)	19(65.5)	69(63.3)	
G3 + G2	50(36.2)	10(34.5)	40(36.7)	

T_is_, severe dysplasia; T_1_, indicates the localization of tumor cells in the esophageal tunica mucosa; T_2_, indicates the localization of tumor cells in esophageal muscular layer; T_3_ indicates the invasion of tumor cells in esophageal adventitia; G1, well differentiation; G2, moderate differentiation; G3, poor differentiation

**Table 2:** Cox regression model analyze the factors of affecting prognosis

Variable	β	*χ*2	*p* Value	HR	95% CI	
					Lower	Upper
Gender	−0.135	0.210	0.647	0.874	0.490	1.557
Age (year)	−0.008	0.361	0.548	0.992	0.965	1.019
Depth of invasion	0.382	5.557	0.018	1.465	1.066	2.011
Lymph node metastasis	−0.081	0.052	0.820	0.922	0.457	1.860
TNM Stage	−0.312	2.665	0.103	0.732	0.504	1.064
Histological grade	−0.298	2.207	0.137	0.742	0.501	1.100
CR-1 expression	1.180	11.699	0.001	3.253	1.655	6.395

### CR-1 is co-expressed with ALDH1A1 by ESCC cells

**Fig. 5:**
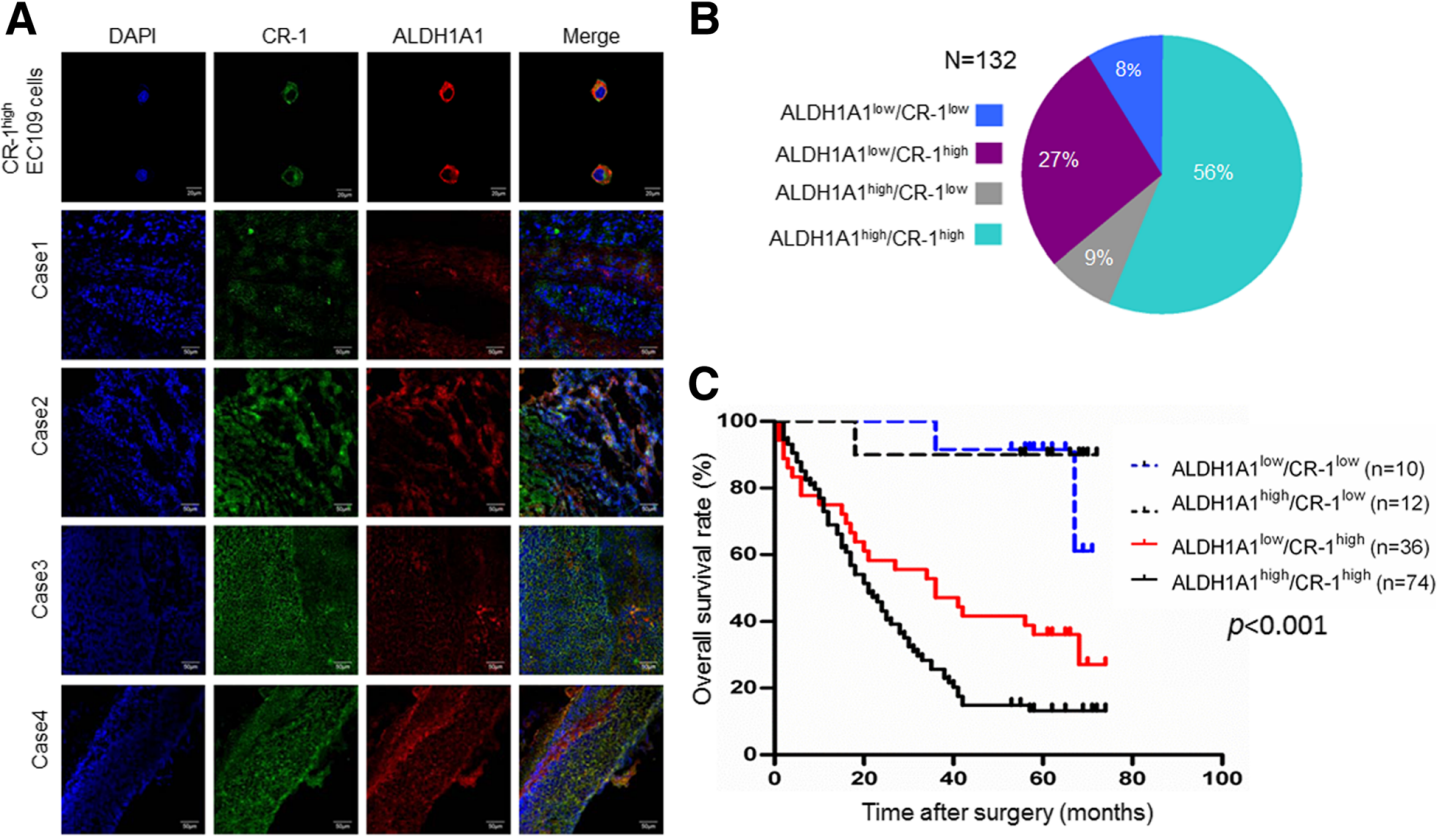
Co-expression of CR-1 and ALDH1A1 and prognostic significance in ESCC patients. **a** Confocal microscopic analysis of co-expression of CR-1 and ALDH1A1 in EC109 cells and ESCC specimens from four patients. **b** Quantitative analysis of the percentage of co-expression of CR-1/ALDH1A1 in 132 patients. **c** Kaplan-Meier analysis indicates that co-expression of CR-1 and ALDH1A1 predicts the shortest survival in ESCC patients

We previously evidenced that ALDH1A1^high^ ESCC cells possess CSLC properties and contribute to the poor prognosis of human ESCC [4]. Hence, we examined the co-expression between CR-1 and ALDH1A1 in cells, frozen and serial paraffin sections of ESCC. Laser confocal microscopy showed that CR-1 expression was overlapped with ALDH1A1 in ESCC cells and in frozen sections (Fig. 5a). As shown in Fig. 5b, the percentage of overlapping of CR-1 expression with ALDH1A1^high^ was 56% (*n* = 74) in 132 ESCC cases detected by IHC staining on serial paraffin sections of ESCC. Survival curves of 132 patients were analyzed using the Kaplan-Meier method. A significant correlation was found between the staining of ALDH1A1^high^/CR-1^high^ and decreased overall survival in ESCC patients (*p* < 0.001, Fig. 5c). Our results suggest that there is a good overlap between CR-1^high^ ECSLCs and ALDH1A1^high^ ECSLCs, therefore CR-1 together with ALDH1A1 can be applied as useful biomarkers to predict the outcome of ESCC patients.

## Discussion

Recently, considerable efforts have been made in the discovery and characterization of CSLC markers. Most markers for sorting CSLC are empirical and derived from normal stem cells, which have been questioned for their specificity and reliability as CSLC markers. Up to now, several molecules have been proposed to be used as markers to characterize the ECSLCs. Neurotrophin receptor p75 (p75NTR), also known as CD271, is a stem cell marker for normal oesophageal epithelial cells [23]. In ESCC cells, Huang et al. [6] demonstrated that p75NTR-positive cells possess some characteristics of CSCs, namely, self-renewal and chemotherapy resistance. Recently, Yamaguchi et al. [24] further demonstrated that p75NTR-positive cells express higher stem cell-related genes (Nanog, p63 and Bmi-1) and EMT-related genes (N-cadherin and fibronectin), exhibite higher abilities of the colony formation in vitro and the tumor formation in vivo than that of CD44 or CD90-positive cells. Also, they found that p75NTR-positive/CD44-negative and p75NTR-positive/CD90-negative cell fractions contain significantly higher proportions of mitotically quiescent cells. Hence, p75NTR expression may serve as a characteristic of the mitotically quiescent cancer stem cell population present in ESCC. With two primary ESCC cells, Zhao et al. [5] selected ECSLC markers from the candidates that had been used in other solid carcinomas, including CD44, CD90, CD133, CD271 and CD326. They found that only CD44 expression is correlated with tumorigenicity. Aldehyde dehydrogenase 1A1 (ALDH1A1), an intracellular enzyme responsible for detoxifying aldehydes, is a cancer stem-like cell-associated protein in various malignant [25]. Almanaa et al. [26] reported that ALDH1-positive esophageal cancer cells have high cell proliferation rates and the ability to regenerate tumor bulk. Our previous work [4] demonstrated that ALDH1A1-positive ESCC cells possess properties of cancer stem-like cells and highly invasive potential. CD90, also called Thy-1 (thymocyte differentiation factor 1), has been identified in various stem cells and CSLCs [27]. Tang et al. [8] reported that CD90-positive ESCC cells exhibit stem cell-like features and have high tumourigenic and metastatic capacities. Thus it can be seen that there are many inconsistencies and contradictions among these markers for characterization of ECSCs. To circumvent these challenging issues, more functional ECSLC markers should be exploited. Accordingly, CR-1 showed promising potential as a functional marker for the identification and isolation of CSLC from ESCC. Here, we demonstrated that CR-1^high^ ESCC cells express high level of stemness-related genes, and possess high potential of self-renewal and high ability of tumorigenesis. Silencing CR-1 expression significantly reduced the expression of stemness-related genes and abilities of self-renewal and tumorigenesis. We also evaluated the consistency between CR-1 expression with ALDH1 expression, and found that these two markers are highly overlapped in ESCC cell lines and 64% (85/132) cases are consistent in ESCC specimens. But the possible overlapping of CR-1 expression with that of CD90, CD44, p75^NTR^ in ECSLCs requires further investigation. In addition, antisense nucleic acid technique on CR-1 [28], and antagonist of the CR-1 CFC domain have been reported to inhibit human breast cancer, colon cancer and testicular cancer cells growth [29], suggesting that CR-1 is a valuable therapeutic target for targeting CSLCs in ESCC.

EMT describes a mechanism by which cells lose their epithelial characteristics and acquire more migratory mesenchymal properties. In the context of cancer, EMT facilitates the dissemination of cancer cells and endows them with properties essential for metastasis including stemness, invasiveness, and the ability to survive in the circulation and seed at a secondary site [22, 30–32]. Indeed, a number of epigenetic regulators are known to functionally regulate genes important for EMT [33]. Despite a few reported EMT-related molecules in ESCC [34–39], the molecular mechanisms regulating EMT in ESCC remain elusive. Results from the current study suggest that CR-1 regulates EMT in ESCC as knockdown of CR-1 in EC109 and TE-1 cells reversed EMT as well as the invasive and metastatic properties of the cells. EMT has been shown to be associated with early steps of invasion and metastasis of epithelial origin cancer cells [40]. These results are consistent with the previous reports that CR-1 is crucial for the activation of EMT in some cancers, including ESCC [41–45]. Increasing evidence has suggested that EMT generates cells with properties of CSLC cells [32], and our results demonstrate that EMT promoted by CR-1 is involved in generation of ECSLCs.

To emphasize the role of CR-1 in ESCC, we analyzed CR-1 levels in clinical ESCC specimens. We demonstrated that the most ESCCs had positive CR-1 expression, which is closely related to the depth of tumor invasion, lymph node metastasis and poor prognosis in the ESCC patients. Metastasis and invasion are the major causes of poor prognosis for ESCC patients. At present, the Tumor, Node, Metastasis (TNM) staging system is the primary tool to determine the extent of cancer and the prognosis of patients, and functions as a surrogate for survival. However, due to the existence of undetectable micrometastasis and low sensitivity of clinical imaging, this system does not always predict prognosis accurately. Discovering new molecular markers, related to the metastasis and invasion, is a promising strategy to achieve more accurate clinical outcome predictions and treatment options for ESCC. Our results suggest that CR-1 can serve as such a marker for ESCC, which is consistent with the previous reports in cervical carcinoma [46], gastric cancer [47], and non-small cell lung cancer [48].

## Conclusions

In summary, this study illustrates that CR-1 acts as a functional ECSLC marker and can be used as a malignant prognostic indicator in ESCC patients. CR-1 together with ALDH1A1 can be applied as useful biomarkers to predict the outcome of ESCC patients. The potential role of CR-1 as a therapeutic target for the development of novel ESCC therapeutics targeting ECSLCs needs to be further investigated.

## Additional file

Additional file 1: Supplementary materials and methods, including Quantitative RT-PCR, Western blotting, Flow cytometry, Silencing CR-1 with shRNA, and Immunofluorescence staining. **Table S1.** Primer sequences for qRT-PCR assay. **Table S2.** shRNA sequences targeting CR-1. **Table S3.** Incidence of tumor formation in nude mice injected with ESCC cells. **Table S4.** The frequency of lung metastasis (1x10^4^ cells/mouse). **Table S5.** Significant difference of CR-1 expression between carcinoma and adjacent normal tissues. **Figure S1.** The levels of CR-1expression and the silencing efficiency of CR-1 shRNA in ESCC cells. **Figure S2.** Silencing CR-1 expression significantly represses the self-renewal and tumorigenicity inTE-1 cells. **Figure S3.** Suppression of CR-1 expression inhibits the invasive and metastatic capabilities of TE-1 cells in vitro and in vivo. **Figure S4.** The effect of silencing CR-1 on the expression of MMPs in EC109 cells. (DOC 1602 kb)

## Abbreviations

ALDH1A1: Aldehyde dehydrogenase 1A1
CR-1: Cripto-1
CSLCs: Cancer stem-like cells
ECSLCs: ESCC cancer stem-like cells
EMT: Epithelial-mesenchymal transition
ESCC: Esophageal squamous cell carcinoma
FACS: Fluorescence activated cell sorting
IHC: Immunohistochemistry
MMPs: Matrix metalloproteinases
shRNA: Short hairpin RNA
TDGF-1: Teratoma-derived growth factor 1

## References

[1] Chen W, Zheng R, Baade PD (2016). Cancer statistics in China, 2015. CA Cancer J Clin.

[2] Zhang HZ, Jin GF, Shen HB (2012). Epidemiologic differences in esophageal cancer between Asian and Western populations. Chin J Cancer.

[3] Clarke MF, Dick JE, Dirks PB (2006). Cancer stem cells--perspectives on current status and future directions: AACR Workshop on cancer stem cells. Cancer Res.

[4] Yang L, Ren Y, Yu X (2014). ALDH1A1 defines invasive cancer stem-like cells and predicts poor prognosis in patients with esophageal squamous cell carcinoma. Mod Pathol.

[5] Zhao JS, Li WJ, Ge D (2011). Tumor initiating cells in esophageal squamous cell carcinomas express high levels of CD44. PLoS One.

[6] Huang SD, Yuan Y, Liu XH (2009). Self-renewal and chemotherapy resistance of p75NTR positive cells in esophageal squamous cell carcinomas. BMC Cancer.

[7] Okumura T, Tsunoda S, Mori Y (2006). The biological role of the low-affinity p75 neurotrophin receptor in esophageal squamous cell carcinoma. Clin Cancer Res.

[8] Tang KH, Dai YD, Tong M (2013). A CD90(+) tumor-initiating cell population with an aggressive signature and metastatic capacity in esophageal cancer. Cancer Res.

[9] Ciccodicola A, Dono R, Obici S (1989). Molecular characterization of a gene of the ‘EGF family’ expressed in undifferentiated human NTERA2 teratocarcinoma cells. EMBO J.

[10] Rangel MC, Karasawa H, Castro NP (2012). Role of Cripto-1 during epithelial-to-mesenchymal transition in development and cancer. Am J Pathol.

[11] Watanabe K, Meyer MJ, Strizzi L (2010). Cripto-1 is a cell surface marker for a tumorigenic, undifferentiated subpopulation in human embryonal carcinoma cells. Stem Cells.

[12] Mancino M, Strizzi L, Wechselberger C (2008). Regulation of human Cripto-1 gene expression by TGF-beta1 and BMP-4 in embryonal and colon cancer cells. J Cell Physiol.

[13] Pilgaard L, Mortensen JH, Henriksen M (2014). Cripto-1 expression in glioblastoma multiforme. Brain Pathol.

[14] Wang JH, Wei W, Xu J (2015). Elevated expression of Cripto-1 correlates with poor prognosis in hepatocellular carcinoma. Oncotarget.

[15] Castro NP, Fedorova-Abrams ND, Merchant AS (2015). Cripto-1 as a novel therapeutic target for triple negative breast cancer. Oncotarget.

[16] Xu CH, Cao L, Wei Y (2015). Serum cripto-1 as a clinical marker for lung cancer. Int J Biol Markers.

[17] Francescangeli F, Contavalli P, De Angelis ML (2015). Dynamic regulation of the cancer stem cell compartment by Cripto-1 in colorectal cancer. Cell Death Differ.

[18] Strizzi L, Margaryan NV, Gilgur A (2013). The significance of a Cripto-1 positive subpopulation of human melanoma cells exhibiting stem cell-like characteristics. Cell Cycle.

[19] Wu F, Zhou Q, Yang J (2011). Endogenous axon guiding chemorepulsant semaphorin-3 F inhibits the growth and metastasis of colorectal carcinoma. Clin Cancer Res.

[20] Camp RL, Dolled-Filhart M, Rimm DL (2004). X-tile: a new bio-informatics tool for biomarker assessment and outcome-based cut-point optimization. Clin Cancer Res.

[21] Fabregat I, Malfettone A, Soukupova J. New Insights into the Crossroads between EMT and Stemness in the Context of Cancer. J Clin Med. 2016;5(3). 10.3390/jcm5030037 PMC4810108 26985909

[22] Thiery JP (2002). Epithelial-mesenchymal transitions in tumour progression. Na Rev Cancer.

[23] Okumura T, Shimada Y, Imamura M, Yasumoto S (2003). Neurotrophin receptor p75(NTR) characterizes human esophageal keratinocyte stem cells in vitro. Oncogene.

[24] Yamaguchi T, Okumura T, Hirano K, Watanabe T, Nagata T, Shimada Y, Tsukada K (2016). p75 neurotrophin receptor expression is a characteristic of the mitotically quiescent cancer stem cell population present in esophageal squamous cell carcinoma. Int J Oncol.

[25] Xu X, Chai S, Wang P (2015). Aldehyde dehydrogenases and cancer stem cells. Cancer Lett.

[26] Almanaa TN, Geusz ME, Jamasbi RJ (2013). A new method for identifying stem-like cells in esophageal cancer cell lines. J Cancer.

[27] Kumar A, Bhanja A, Bhattacharyya J, Jaganathan BG. Multiple roles of CD90 in cancer. Tumour Biol. 2016;37(9):11611–22. 10.1007/s13277-016-5112-0 27337957

[28] Normanno N, Tortora G, De Luca A (1999). Synergistic growth inhibition and induction of apoptosis by a novel mixed backbone antisense oligonucleotide targeting CRIPTO in combination with C225 anti-EGFR monoclonal antibody and 8-Cl-cAMP in human GEO colon cancer cells. Oncol Rep.

[29] Adkins HB, Bianco C, Schiffer SG (2003). Antibody blockade of the Cripto CFC domain suppresses tumor cell growth in vivo. J Clin Invest.

[30] Kalluri R, Weinberg RA (2009). The basics of epithelial-mesenchymal transition. J Clin Invest.

[31] Polyak K, Weinberg RA (2009). Transitions between epithelial and mesenchymal states: acquisition of malignant and stem cell traits. Nat Rev Cancer.

[32] Mani SA, Guo W, Liao MJ (2008). The epithelial-mesenchymal transition generates cells with properties of stem cells. Cell.

[33] Wu CY, Tsai YP, Wu MZ (2012). Epigenetic reprogramming and post-transcriptional regulation during the epithelial-mesenchymal transition. Trends Genet.

[34] Min S, Xiaoyan X, Fanghui P (2013). The glioma-associated oncogene homolog 1 promotes epithelial--mesenchymal transition in human esophageal squamous cell cancer by inhibiting E-cadherin via Snail. Cancer Gene Ther.

[35] Wang Q, Ma C, Kemmner W (2013). Wdr66 is a novel marker for risk stratification and involved in epithelial-mesenchymal transition of esophageal squamous cell carcinoma. BMC Cancer.

[36] Yokobori T, Suzuki S, Tanaka N (2013). MiR-150 is associated with poor prognosis in esophageal squamous cell carcinoma via targeting the EMT inducer ZEB1. Cancer Sci.

[37] Cheung PY, Yip YL, Tsao SW (2011). Id-1 induces cell invasiveness in immortalized epithelial cells by regulating cadherin switching and Rho GTPases. J Cell Biochem.

[38] Natsuizaka M, Ohashi S, Wong GS (2010). Insulin-like growth factor-binding protein-3 promotes transforming growth factor-{beta}1-mediated epithelial-to-mesenchymal transition and motility in transformed human esophageal cells. Carcinogenesis.

[39] Rees JR, Onwuegbusi BA, Save VE (2006). In vivo and in vitro evidence for transforming growth factor-beta1-mediated epithelial to mesenchymal transition in esophageal adenocarcinoma. Cancer Res.

[40] Boyer B, Valles AM, Edme N (2000). Induction and regulation of epithelial-mesenchymal transitions. Bioche Pharmacol.

[41] Shibamoto S, Hayakawa M, Takeuchi K (1994). Tyrosine phosphorylation of beta-catenin and plakoglobin enhanced by hepatocyte growth factor and epidermal growth factor in human carcinoma cells. Cell Adhes Commun.

[42] Ebert AD, Wechselberger C, Nees M (2000). Cripto-1-induced increase in vimentin expression is associated with enhanced migration of human Caski cervical carcinoma cells. Exp Cell Res.

[43] Strizzi L, Bianco C, Normanno N (2004). Epithelial mesenchymal transition is a characteristic of hyperplasias and tumors in mammary gland from MMTV-Cripto-1 transgenic mice. J Cell Physiol.

[44] Bianco C, Strizzi L, Ebert A (2005). Role of human cripto-1 in tumor angiogenesis. J Natl Cancer Inst.

[45] Huang C, Chen W, Wang X (2015). Cripto-1 Promotes the Epithelial-Mesenchymal Transition in Esophageal Squamous Cell Carcinoma Cells. Evid Based Complement Alternat Med.

[46] Ertoy D, Ayhan A, Sarac E (2000). Clinicopathological implication of cripto expression in early stage invasive cervical carcinomas. Eur J Cancer.

[47] Zhong XY, Zhang LH, Jia SQ (2008). Positive association of up-regulated Cripto-1 and down-regulated E-cadherin with tumour progression and poor prognosis in gastric cancer. Histopathology.

[48] Xu CH, Sheng ZH, Hu HD (2014). Elevated expression of Cripto-1 correlates with poor prognosis in non-small cell lung cancer. Tumour Biol.

