# Supplementary material for: Cripto-1 acts as a functional marker of cancer stem-like cells and predicts prognosis of the patients in esophageal squamous cell carcinoma
Source: Mol Cancer. 2017 Apr 21;16:81. doi: 10.1186/s12943-017-0650-7 (PMC5399850; doi:10.1186/s12943-017-0650-7)
Supplement: Supplementary file 1 — Supplementary materials and methods, including Quantitative RT-PCR, Western blotting, Flow cytometry, Silencing CR-1 with shRNA, and Immunofluorescence staining. Table S1. Primer sequences for qRT-PCR assay. Table S2. shRNA sequences targeting CR-1. Table S3. Incidence of tumor formation in nude mice injected with ESCC cells. Table S4. The frequency of lung metastasis (1x10^4^ cells/mouse). Table S5. Significant difference of CR-1 expression between carcinoma and adjacent normal tissues. Figure S1. The levels of CR-1expression and the silencing efficiency of CR-1 shRNA in ESCC cells. Figure S2. Silencing CR-1 expression significantly represses the self-renewal and tumorigenicity inTE-1 cells. Figure S3. Suppression of CR-1 expression inhibits the invasive and metastatic capabilities of TE-1 cells in vitro and in vivo. Figure S4. The effect of silencing CR-1 on the expression of MMPs in EC109 cells. (DOC 1602 kb) [file 12943_2017_650_MOESM1_ESM.doc]

**SupplementaryMeterials and Methods**

*Quantitative RT-PCR*

Total RNAs were extracted from the tissue samples and cell lines by using TRIzol™ reagent (TakaRa, Japan), and were then reversely transcribed into cDNA with RT kit (TakaRa). The levels of targeted mRNA expression were measured by real-time quantitative PCR (qRT-PCR). A housekeeping gene β-actin was used as an internal control. Primers used in this study were listed in supplementary information Table S1. All of qRT-PCR experiments were repeated at least three times.

*Western blotting*

Cells were harvested and washed twice with ice-cold PBS and lysed on ice with protein extraction reagent (Beyotime Company, China) supplemented with 1% protease inhibitor (Pierce, Rockford, IL). Lysed samples were then centrifuged at 12,000×g (4°C for 20 min). Supernatant was collected and the protein concentration was determined using BCA protein assay kit (Pierce). Total protein was separated by 10% SDS-polyacrylamide gel electrophoresis (SDS-PAGE) and was then transferred to polyvinylidene fluoride membranes (Millipore, Billerica, MA).Membranes were treated for 2 hrs at room temperature in PBS containing 5% bovine serum albumin (Sigma-Aldrich, St. Louis, MO) and 0.1% Tween-20, and then incubated at 4°C overnight with following primary antibodies: polyclonal rabbitanti-CR-1(1:500, ab19917, Abcam, Cambridge, UK),monoclonal rabbit anti-E-cadherin (1:500, Cell Signaling Technology, Beverly, MA), monoclonal mouse anti-snail (1:500, Cell Signaling Technology), polyclonal rabbit anti-MMP9(1:600, Cell Signaling Technology), polyclonal rabbit anti-vimentin (1:500, Cell Signaling Technology), monoclonal rabbit anti β-actin (1:1000, Cell Signaling Technology). After washed 3 times with PBS containing 0.1% Tween-20, the membranes were incubated with peroxidase conjugated secondary antibody (1:1000, Beyotime Company) for one hour. Then chemiluminescence was detected using SuperSignal West Femto Maximum Sensitivity Substrate (Pierce).

*Flow cytometry*

Cell suspension was stained with a rabbit anti-human CR-1 antibody (Novus Biologicals, Littleton, CO). Alexa Fluor 488-labeled donkey anti-rabbit IgG (H+L) antibody (Invitrogen) was used as secondary antibody. Flow cytometric analysis or sorting was carried out with BD FACS Calibur or FACS Aria^TM^ II (BD Biosciences, Franklin Lakes, NJ). Data were analyzed by using FlowJo 7.6 software (Tree Star, Ashland, OR).

*Silencing CR-1 with shRNA*

Three self-complementary hairpin DNA fragments targeting different region of CR-1 mRNA and a negative mock control were synthesized and cloned into pMAGic 7.1, and named as Lentivirus/shRNA-CR1 (shCR1) and Lentivirus/shRNA-mock (Mock), respectively. Viral packaging was performed by co-transfection of HEK293T cells with these plasmids using Lipofectamine 2000™ (Invitrogen) and polybrene (Santa Cruz Biotechnology, Santa Crutz, CA) in serum-free DMEM. Culture supernatants containing lentiviral particles were collected at 24 and 48 hours after transfection, pooled and filtered through a 0.45µm membrane to remove cell debris. Viral particles were concentrated by precipitation with PEG-it Virus precipitation solution (System Biosciences, Mountain View, CA) according to manufacturer’s instructions. To gain silencing CR-1 cells, ESCC cells were grown to 70-80% confluence and infected with shCR1 and Mock at MOI of 10 in the presence of 3 µg/ml polybrene. Green fluorescence protein (GFP) positive cells were selected by flow cytometry. Three sequences of the short hairpin RNA targeting CR-1 were available in supplementary information Table S2. The efficacy of silencing of CR-1 gene was assessed by western blotting.

*Immunoﬂuorescence staining*

Analysis for differentiation of ECSLCs was performed with mouse anti-human CK18 antibody (1:100, Beijing Zhongshan Jinqiao Company, China) as described previously. For co-expression analysis of CR-1 and ALDH1A1in cell lines, cultured cells were fixed in 4%paraformaldehyde. Primary polyclonal rabbitanti-humanCR-1antibody (1:100, ab19917, Abcam, UK), mouse anti-human ALDH1A1antibody (1:100, BD Biosciences) were used. For tissue experiments, human ESCC samples were frozen and fixed in cold acetone. Primary anti-human CR-1 and anti-human ALDH1A1 antibodies were same as above. After being labeled with the corresponding secondary antibodies, all samples were then examined under a laser confocal scanning microscope (SP-5, Leica, Germany).

**Supplementary Tables**

**Table S1.** Primer sequences for qRT-PCR assay

| Genes | Primers sequences | Production size (bp) |
| --- | --- | --- |
| CR-1 | F: GATACAGCACAGTAAGGAGC  R: TAGTTCTGGAGTCCTGGAAG | 286 |
| Oct-4 | F: GCAGCGACTATGCACAACGA  R: CCAGAGTGGTGACGGAGACA | 195 |
| Sox-2 | F: CATCACCCACAGCAAATGACA  R:GCTCCTACCGTACCACTAGAACTT | 242 |
| Nanog | F: CTCTCCAACATCCTGAACCTC  R: GGTTCCCAGTCGGGTTCAC | 196 |
| Vimentin | F: GGGACCTCTACGAGGAGGAG  R: CGCATTGTCAACATCCTGTC | 200 |
| E-cadherin | F: CGAGAGCTACACGTTCACGG  R: GGGTGTCGAGGGAAAAATAGG | 200 |
| N-cadherin | F: AGCCAACCTTAACTGAGGAGT  R: GGCAAGTTGATTGGAGGGATG | 136 |
| β-actin | F: TTGCGTTACACCCTTTCTTG  R: CACCTTCACCGTTCCAGTTT | 147 |
| SNAI1/Snail1 | F: TCGGAAGCCTAACTACAGCGA  R: AGATGAGCATTGGCAGCGAG | 140 |
| SNAI2/Snail2(Slug) | F: TGTGACAAGGAATATGTGAGCC  R: TGAGCCCTCAGATTTGACCTG | 203 |
| Twist | F: GTCCGCAGTCTTACGAGGAG  R: GCTTGAGGGTCTGAATCTTGCT | 156 |
| [ZEB1](http://en.wikipedia.org/wiki/ZEB1) | F: GATGATGAATGCGAGTCAGATGC  R: ACAGCAGTGTCTTGTTGTTGT | 86 |
| ZEB2 | F: GGAGACGAGTCCAGCTAGTGT  R: CCACTCCACCCTCCCTTATTTC | 107 |
| E47 | F: ACGAGCGTATGGGCTACCA  R: GTTATTGCTTGAGTGATCCGGG | 233 |
| [SIX1](http://en.wikipedia.org/wiki/SIX1) | F: CTGCCGTCGTTTGGCTTTAC  R: GCTCTCGTTCTTGTGCAGGT | 135 |
| [FOXC2](http://en.wikipedia.org/wiki/FOXC2) | F: CCTCCTGGTATCTCAACCACA  R: GAGGGTCGAGTTCTCAATCCC | 134 |
| MMP2 | F: CCACTGCCTTCGATACAC  R: GAGCCACTCTCTGGAATCTTAAA | 133 |
| MMP7 | F: GAGTGAGCTACAGTGGGAACA  R: CTATGACGCGGGAGTTTAACAT | 158 |
| MMP9 | F: GTTCCCGGAGTGAGTTGA  R: TTTACATGGCACTGCCAAAGC | 110 |

**Table S2.** shRNA sequences targeting CR-1

| No. | shRNA sequences targeting CR-1 (Gene ID: 6997) |
| --- | --- |
| sh1 | 5’-CGCUUCUCUUACAGUGUGA-3’ |
| sh2 | 5’-GCTAAATGGAAGGGCAAGTTT-3’ |
| sh3 | 5’-ACAGCACAGTAAGGAGCTAAA-3’ |
| Control | 5’-TTCTCCGAACGTGTCACGT-3’ |

**Table S3.** Incidence of tumor formation in nude mice injected with ESCC cells

|  | Cell number | Mock | shCR1 | *p* Value |
| --- | --- | --- | --- | --- |
| EC109 | 2×10^4^ | 5/5 | 2/5 | <0.05 |
|  | 2×10^5^ | 5/5 | 5/5 |  |
| TE-1 | 2×10^4^ | 5/5 | 2/5 | <0.05 |
|  | 2×10^5^ | 5/5 | 5/5 |  |

**Table S4.** The frequency of lung metastasis (1x10^4^ cells/mouse)

|  | Mock | ShCR1 | p Value |
| --- | --- | --- | --- |
| EC109 | 4/6 | 0/6 | 0.030 |
| TE-1 | 5/6 | 1/6 | 0.040 |

**Table S5.** Significant difference of CR-1 expression between carcinoma and adjacent normal tissues

| Samples | Case | Expression of CR-1 | | *p* Value |
| --- | --- | --- | --- | --- |
|  |  | CR-1^low^ (%) | CR-1^high^ (%) |  |
| Adjacent normal tissues | 138 | 118 (85.5) | 20 (14.5) | <0.001 |
| Carcinoma tissues | 138 | 42 (30.4) | 96 (69.6) |  |

**Supplementary Figures**

**
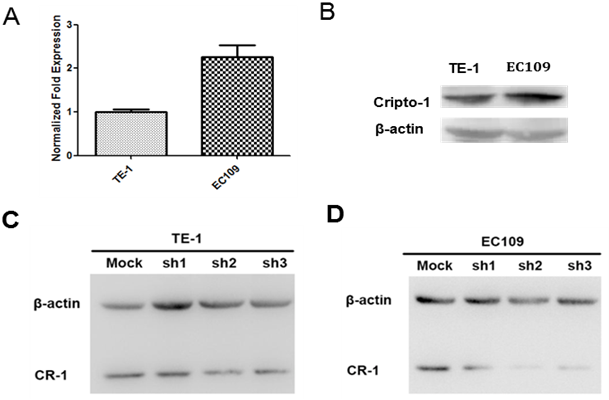
**

**Figure S1.** The levels of CR-1expression and the silencing efficiency of CR-1 shRNA in ESCC cells.

A. The expression of CR-1 in TE-1 and EC109 cells was detected by qRT-PCR. The transcript levels were normalized against GAPDH. B. The protein abundance of CR-1 in TE-1 and EC109 cells estimated by western blotting. C and D. The silencing efficiency of CR-1 expression with shRNA evaluated by western blotting in TE-1 (C) and EC109 (D) cells.


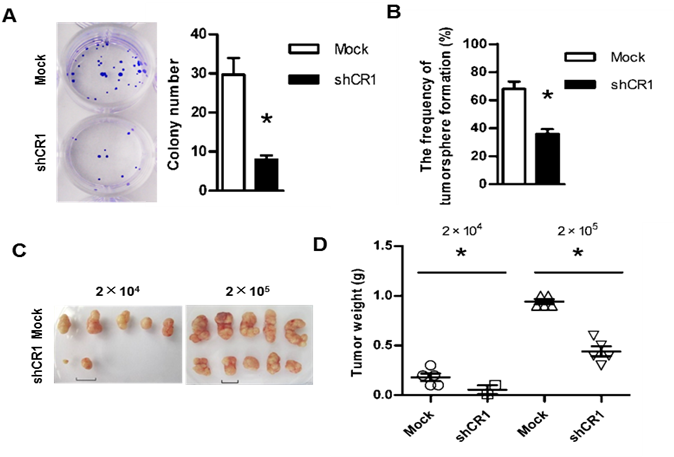


**Figure S2.** Silencing CR-1 expression significantly represses the self-renewal and tumorigenicity inTE-1 cells.

A. Knockdown of CR-1 expression dramatically reduced the capability of colony formation. B. Silencing CR-1 expression significantly inhibited the frequency of sphere formation. C. Images of xenograft tumors showed that CR-1 knockdown lowered tumor formation rate and tumor size in TE-1 cells. D. The weight of xenografts derived from shCR1 TE-1 cells was significantly lighter than that derived from mock cells.


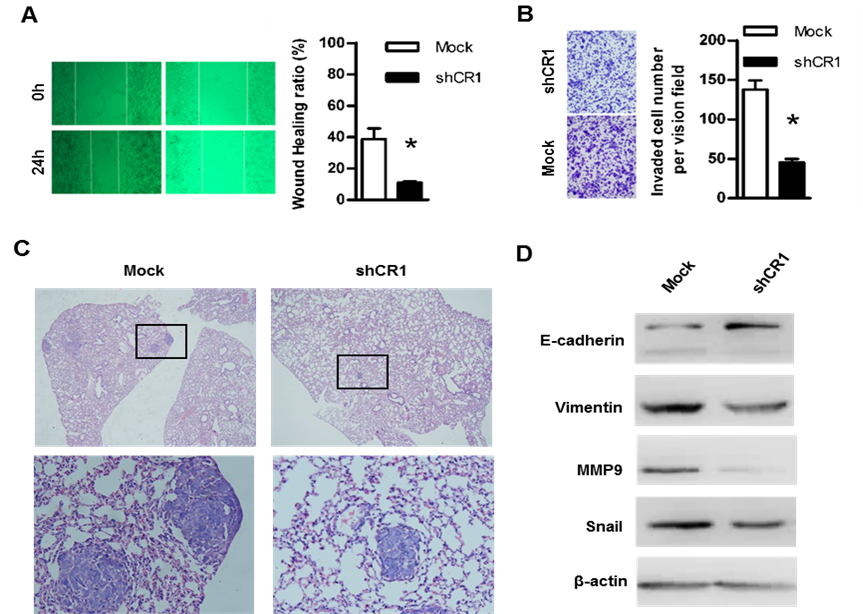


**Figure S3.** Suppression of CR-1 expression inhibits the invasive and metastatic capabilities of TE-1 cells *in vitro* and *in vivo.*

A. Wound healing experiments showed that silencing CR-1 expression significantly suppressed the migration of TE-1 cells. B. Silencing CR-1 expression decreased the invasive ability of TE-1 cells *in vitro*. C. Lung metastasis model of nude mice showed that CR-1knockdown suppressed the metastasis of TE-1 cells. D. Western blotting assay showed that CR-1 knockdown induced the up-regulation of E-cadherin and the down-regulation of vimentin, snail and MMP9 in TE-1 cells. * indicates *p*< 0.05.

**
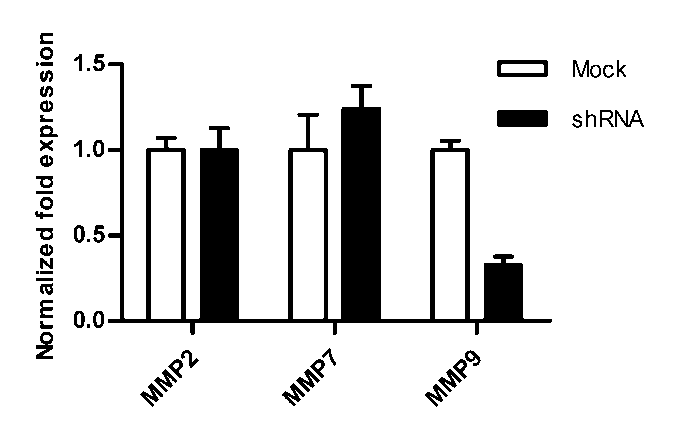
**

*

**Figure S4.**The effect of silencing CR-1 on the expression of MMPs in EC109 cells.

qRT-PCR analysis showed that CR-1 knockdown markedly down-regulated the expression of MMP9 at mRNA level in EC109 cells, but not MMP2 and MMP7.

**Liu Q, et al. Supplementary file**
